# Alcohol abrogates human norovirus infectivity in a pH-dependent manner

**DOI:** 10.1038/s41598-020-72609-z

**Published:** 2020-09-28

**Authors:** Shintaro Sato, Naomi Matsumoto, Kota Hisaie, Satoshi Uematsu

**Affiliations:** 1 Mucosal Vaccine Project, BIKEN Innovative Vaccine Research Alliance Laboratories, Research Institute for Microbial Diseases, Osaka University, Osaka, 565-0871 Japan; 2 Department of Virology, Research Center for Infectious Disease Control, Research Institute for Microbial Diseases, Osaka University, Osaka, 565-0871 Japan; 3 Department of Immunology and Genomics, Osaka City University Graduate School of Medicine, Osaka, 545-8585 Japan

**Keywords:** Virology, Public health

## Abstract

Alcohol-based disinfectants are widely used for the sanitization of microorganisms, especially those that cause infectious diseases, including viruses. However, since the germicidal mechanism of alcohol is lipolysis, alcohol-based disinfectants appear to have a minimal effect on non-enveloped viruses, such as noroviruses. Because there is no cultivation method for human norovirus (HuNoV) in vitro, murine norovirus and feline calicivirus have been used as surrogates for HuNoV to analyze the efficacy of disinfectant regents. Therefore, whether these disinfectants and their conditions are effective against HuNoVs remain unknown. In this study, we report that ethanol or isopropanol alone can sufficiently suppress GII.4 genotype HuNoV replication in human iPSC-derived intestinal epithelial cells. Additionally, pH adjustments and salting-out may contribute toward the virucidal effect of alcohol against other HuNoV genotypes and cancel the impediment of organic substance contamination, respectively. Therefore, similar to sodium hypochlorite, alcohol-based disinfectants containing electrolytes can be used for HuNoV inactivation.

## Introduction

Alcohols are widely used as disinfectants for sanitization, especially for skin surfaces, cooking devices, tableware, and high-touch surfaces, such as doorknobs, balustrades, and elevator buttons in hospitals, other healthcare settings, and households. Among the several types of alcohol, ethanol and isopropanol are preferably used as disinfectants. Although the molecular mechanisms underlying the bactericidal effect of alcohol are not fully understood, high-percentage (around 70% [v/v]) ethanol shows a polymer-like structure with water through the formation of hydrogen bonds and hydrophobic bonds, generating a large hydrophobic cluster surface^1^. This hydrophobicity (or lipophilicity) subsequently damages the phospholipid membrane of bacteria by the delipidation and denaturation or coagulation of membrane proteins. Enveloped viruses, such as influenza viruses and coronaviruses, are also susceptible to alcohol-based disinfectants since their envelopes consist of a host lipid bilayer.

Noroviruses belong to a family of non-enveloped RNA viruses and comprise a genetically diverse group whose classification was recently updated based on their complete capsid amino acid sequences and the partial nucleotide sequences of their RNA-dependent RNA polymerases^2^. Since the genogroup of norovirus I, II, and IV infect humans, they are called “human norovirus (HuNoV)”. HuNoVs are the leading cause of intestinal infectious gastroenteritis in people of all ages in developed and developing countries. Therefore, it has major social and economic influences. HuNoV infections can lead to mortality in children, elderly, and immunocompromised patients. Currently, there is no effective vaccine or treatment available. Therefore, if one norovirus patient is in a hospital, nursery school, or aged care facility, spot epidemics of HuNoVs can easily occur via the patient’s vomit and/or fecal excreta. Although it is essential to completely decontaminate HuNoV-contained materials, effective inactivation methods remain to be identified. One of the reasons for this is that there were no in vitro cultivation systems for HuNoVs until 2016^3^. The recent development of a HuNoV replication system in human primary intestinal epithelial cells (IECs) and human induced pluripotent stem cell (iPSC)-derived IECs has spawned advances in HuNoV characterization^4^. Until then, the inactivation methods of HuNoVs were determined using feline calicivirus and/or murine norovirus as surrogates^5–7^. The virucidal efficacy of alcohol and alcohol-based disinfectants for HuNoVs are also considered based on the effects on feline calicivirus or murine norovirus. However, the virucidal efficacy of alcohol for non-enveloped viruses varies in each virus. For example, murine norovirus is sensitive to alcohols, while feline calicivirus is resistant to them^8^.

Although some studies have reported that the level of HuNoV decontamination is determined by viral genome RNA reduction using culture-independent methods^8–10^, it has also been reported that there is no relationship between actual HuNoV propagative activity and the reduction of viral genomes^11^. Therefore, even if viral genome reduction is observed, it is possible that HuNoVs are propagated. The reverse might also be true. Therefore, the virucidal effects of HuNoV disinfectants should be determined by assessing their actual propagative availability.

In the current study, we investigated the virucidal effects of alcohol-based disinfectants for HuNoVs using our in vitro HuNoV propagation method, and found that only ethanol and isopropanol sufficiently suppressed replication of the GII.4 genotype of HuNoV, which causes the majority of the epidemic infections worldwide. In addition, our results suggest that pH-adjusted alcohols, particularly low-pH alcohols, exhibit strong virucidal effects against HuNoVs. Thus, acid-alcohol disinfectants can be associated with good biological safety profiles and can be used for HuNoV inactivation.

## Results

### Virucidal activity of alcohols against HuNoVs

**Figure 1:**
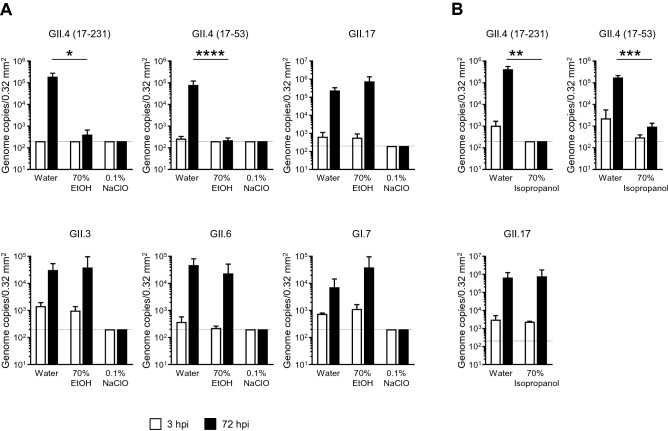
Inactivation of HuNoVs by ethanol, isopropanol, and sodium hypochlorite in suspension. The solutions of the indicated HuNoV genotypes were suspended with threefold volumes of the indicated disinfectants (**A**; 70% ethanol [EtOH] and 0.1% sodium hypochlorite [NaClO], **B**; 70% isopropanol) for 5 min as described in Materials and Methods. Mono-layered human iPSC-derived IECs were inoculated with 2 × 10^6^ (GII.6; 2 × 10^5^, GI.7; 1.8 × 10^6^) genome equivalents of the pre-treated HuNoVs. Inoculation and sampling were performed as described in the Materials and Methods. Viral genome RNA was extracted from both supernatants (i.e., those taken at 3 and 72 h post-infection [hpi]), and then genome equivalents were quantified with RT-qPCR. Samples at 3 hpi were used as references. Each value is representative of at least three independent experiments and is shown as the mean ± SD from 4–6 wells of supernatants for each culture group. *, *P* ≤ 0.05; **, *P* ≤ 0.01; ***, *P* ≤ 0.005; ****, *P* ≤ 0.001. Dashed lines represent the limit of detection.

Although the efficacy of alcohol-based disinfectants against non-enveloped viruses is controversial, whether these disinfectants show virucidal effects on HuNoVs remains to be elucidated. Recently, Costantini et al. demonstrated that 70% (v/v) ethanol or chlorine inactivated GII.4 HuNoV slightly or completely, respectively^11^. Therefore, we first investigated the virucidal effect of 70% ethanol and sodium hypochlorite (NaClO) on several HuNoV genotypes, including GII.4, which is a predominant genotype responsible for HuNoV epidemics. We used a threefold volume of disinfectant solution for the virus solution. Consistent with the previous report, although all the studied HuNoV genotypes were well propagated in our iPSC-derived IECs without any disinfectant condition, a 5 min pretreatment with 0.1% (1000 ppm) NaClO was sufficient to kill all of them (Fig. 1A). However, only 70% ethanol could effectively suppress GII.4 genotype HuNoV replication in our system, but it had no significant effect on the other genotypes tested in this study (Fig. 1A). Similar to 70% ethanol treatment, treatment with 70% isopropanol inhibited the propagative ability of GII.4 but not of GII.17 HuNoV (Fig. 1B). These data indicated that isopropanol could be used as an additive or substituted for ethanol as a GII.4 HuNoV disinfectant.

### Virucidal activity of acid-alcohols against HuNoVs

**Figure 2:**
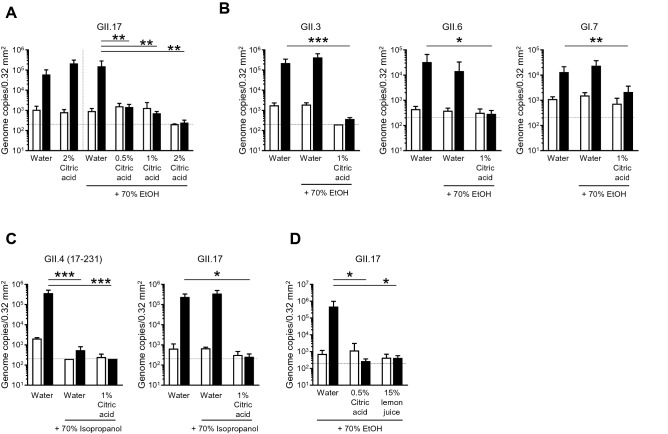
Inactivation of HuNoVs by acid-alcohols in suspension. The solutions of the indicated HuNoV genotypes were suspended with threefold volumes of the indicated disinfectants (**A**, **B**, and **D**; 70% ethanol [EtOH], **C**; 70% isopropanol) for 5 min. Inoculation and sampling were performed as in Fig. 1. Each value is representative of at least three independent experiments and is shown as the mean ± SD from 4–6 wells of supernatants for each culture group. *, *P* ≤ 0.05; **, *P* ≤ 0.01; ***, *P* ≤ 0.005; ****, *P* ≤ 0.001. Dashed lines represent the limit of detection.

It has been reported that low-pH ethanol enhances the virucidal effect on non-enveloped viruses^6^. Therefore, we next tested whether acid–ethanol has potential as a disinfectant for the inactivation of HuNoVs. Similar to ethanol alone, 2% (w/v) citric acid alone (pH is about 1.9) did not influence the propagation of GII.17 HuNoV; however, the addition of at least 0.5% citric acid to ethanol completely inactivated the virus (Fig. 2A). Furthermore, 70% ethanol containing 1% citric acid (pH ~ 3.1) was sufficient for the inactivation of other HuNoV genotypes, such as GII.3, GII.6, and GI.7 (Fig. 2B). In addition, isopropanol could be substituted for ethanol for the acid-alcohol sanitization of HuNoVs (Fig. 2C), indicating that acid-alcohols, similar to NaClO, might be an effective disinfectant for HuNoVs. Pure (100%) lemon juice from concentrate contains about 6% (w/v) citric acid. We examined the virucidal effect of 70% ethanol-15% lemon juice, which contains 1% citric acid, against HuNoV. As shown in Fig. 2D, 70% ethanol-15% lemon juice inhibited GII.17 HuNoV propagation, indicating that concentrated lemon juice can be used as a source of citric acid to generate an acid-alcohol disinfectant.

**Figure 3:**
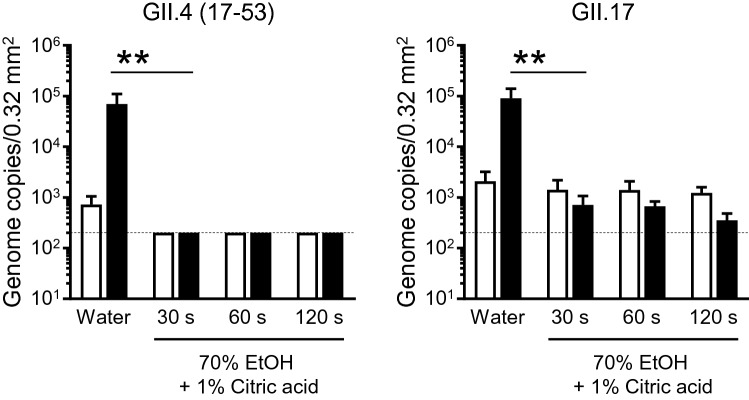
Inactivation of HuNoVs by acid-alcohols in a shorter incubation time. The solutions of the indicated HuNoV genotypes were suspended with threefold volumes of the 70% ethanol [EtOH] plus 1% citric acid for the indicated times. Inoculation and sampling were performed as in Fig. 1. Each value is representative of at least three independent experiments and is shown as the mean ± SD from 4–6 wells of supernatants for each culture group. *, *P* ≤ 0.05; **, *P* ≤ 0.01; ***, *P* ≤ 0.005; ****, *P* ≤ 0.001. Dashed lines represent the limit of detection.

Considering the use of acid–ethanol as a hand sanitizer for HuNoVs, a short incubation time is likely required for inactivation. Therefore, we next examined how much time is needed for the inactivation of HuNoVs with acid–ethanol. In our in vitro HuNoV propagation system, both GII.4 and GII.17 HuNoVs were completely inactivated by 70% ethanol containing 1% citric acid within 30 s of incubation (Fig. 3).

### Virucidal activity of alkaline-alcohol against HuNoVs

**Figure 4:**
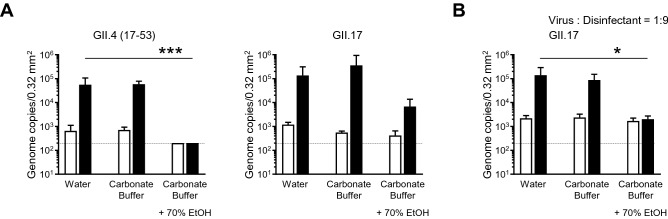
Inactivation of HuNoVs by alkaline-alcohols in suspension. The solutions of the indicated HuNoV genotypes were suspended with threefold (**A**) or ninefold (**B**) volumes of 70% ethanol [EtOH] in carbonate buffer (pH 11) for 5 min. Inoculation and sampling were performed as in Fig. 1. Each value is representative of at least three independent experiments and is shown as the mean ± SD from 4–6 wells of supernatants for each culture group. *, *P* ≤ 0.05; **, *P* ≤ 0.01; ***, *P* ≤ 0.005; ****, *P* ≤ 0.001. Dashed lines represent the limit of detection.

Next, we investigated whether alkaline-alcohol also shows a virucidal effect on HuNoVs. To adjust the pH and generate a weak alkaline (pH ~ 11), we used sodium bicarbonate buffer because both sodium hydrogen carbonate and sodium carbonate are recognized as food additives, and thus its safety has been confirmed similar to citric acid. When HuNoVs were incubated in an alkaline solution (carbonate buffer), both GII.4 and GII.17 HuNoVs were not inactivated to the same extent as that observed in a low-pH solution (Fig. 4A). In addition, a high pH condition did not affect the virucidal effect of 70% ethanol on GII.4 genotype HuNoV (Fig. 4A). Although alkaline-ethanol (carbonate buffer plus 70% ethanol) also appeared to have a virucidal effect on GII.17 HuNoV, which could not be inactivated by 70% ethanol alone, it was less effective than acid–ethanol (Figs. 2A and 4A). However, when we used an increased volume of alkaline-ethanol in a ratio of 9:1 virus solution, GII.17 HuNoV was completely inactivated (Fig. 4B). These data indicate that ethanol, and probably isopropanol, have an actual virucidal effect on HuNoVs that is dependent on the pH.

### Impact of organic substances on the virucidal activity of acid-alcohol against HuNoVs

**Figure 5:**
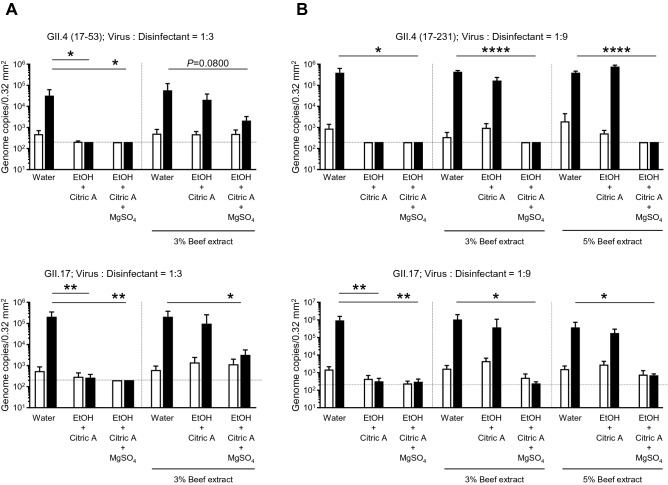
Salting-out efficacy for the contamination of extra organic substances. The solutions of the indicated HuNoV genotypes containing 3% or 5% beef extracts were suspended with threefold (**A**) or ninefold (**B**) volumes of the indicated disinfectants for 5 min. Inoculation and sampling were performed as in Fig. 1. Each value is representative of at least three independent experiments and is shown as the mean ± SD from 4–6 wells of supernatants for each culture group. *, *P* ≤ 0.05; **, *P* ≤ 0.01; ***, *P* ≤ 0.005; ****, *P* ≤ 0.001. Dashed lines represent the limit of detection.

As mentioned above, NaClO has a powerful virucidal effect, even on non-enveloped viruses. However, NaClO also strongly reacts with other organic substances contained in feces and vomit. Therefore, we next investigated the virucidal effect of acid–ethanol on HuNoVs under conditions containing beef extract as an extra-organic substance^12^. Similar to the experiments shown in Fig. 2, GII.4 or GII.17 HuNoV was mixed with acid–ethanol (70% EtOH-1% citric acid). Interestingly, 3% beef extract was sufficient to abolish the virucidal effect of acid–ethanol on GII.4, which could be inactivated by 70% ethanol only (Fig. 5A), indicating that soiled organic substances can inhibit the virucidal activity of acid–ethanol against HuNoVs. As electrolyte solutions, some mineral salts are used to precipitate proteins and low molecular organic compounds, which is a process termed coagulation or salting-out. It is speculated that if organic substances are removed from virus particles, acid–ethanol may inactivate HuNoVs even in a solution containing extra organic substances. In a 3% beef extract solution, 0.05% (w/v) magnesium sulfate (MgSO_4_)-containing acid–ethanol almost completely inactivated the propagation of the GII.17 genotype (Fig. 5A). Although GII.4 HuNoV was also inactivated with MgSO_4_-containing acid–ethanol, it was at a low level, and virus propagation potency was retained. Therefore, we used an increased volume of these disinfectants, similar to the experiments shown in Fig. 4B. A nine times volume of MgSO_4_-containing acid–ethanol but not just acid–ethanol exhibited complete virucidal effects on both GII.4 and GII.17 in a 3% beef extract solution (Fig. 5B). This activity was also maintained in a 5% beef extract solution. These data suggest that MgSO_4_-containing acid–ethanol can be used as a disinfectant for HuNoVs with the same reliability as NaClO, even as hand sanitizer because all of the components in MgSO_4_-containing acid–ethanol are safe for the human body.

### Genomic RNA reduction of acid-alcohol treated HuNoVs

**Figure 6:**
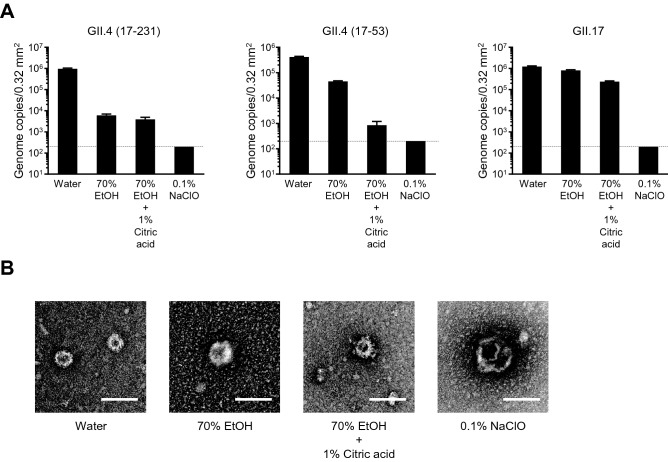
Effects of disinfectants on the genomic RNA and capsid proteins of HuNoVs. (**A**) The solutions of the indicated HuNoV genotypes were suspended with ninefold volumes of the indicated disinfectants for 2 min. Genomic RNA of HuNoVs was prepared and analyzed by qPCR. Each value is representative of three independent experiments and is shown as the mean ± SD of three technical replications. Dashed lines represent the limit of detection. (**B**) GII.17 VLPs were suspended in ninefold volumes of the indicated disinfectants for 2 min. After fixation, transmission electron microscopic analysis was performed. Data are representative of two independent experiments. Scale bar, 100 nm.

Many reports have investigated the effects of alcohol-based disinfectants against murine norovirus and feline calicivirus as surrogates for HuNoV^6,8,13^. Viral genome RNA degradation by alcohol has also been reported in HuNoV^6,9^. When HuNoV was incubated with 0.1% NaClO at room temperature for 2 min, the viral genome could not be detected by real-time PCR (Fig. 6A), indicating that NaClO could disrupt the capsid and/or genome of HuNoVs. In contrast, the genomes of GII.4 and GII.17 HuNoVs could be detected after incubation with 70% ethanol- or citric acid-containing ethanol (Fig. 6A). Although a small decrease in GII.17 genome copies was observed even in acid–ethanol (~ 0.5 log_10_), GII.4 HuNoV was more sensitive to 70% EtOH (~ 2.2 log_10_ for 17–231; 1.0 log_10_ for 17–53) and acid–ethanol (~ 2.4 log_10_ for 17–231; 2.7 log_10_ for 17–53). We further examined the morphological change in virus particles after the incubation with disinfectants. To this end, we used virus-like particles (VLPs), which comprise the VP1 capsid proteins of GII.17^4^. Negative stain transmission electron microscopy analysis revealed that 0.1% NaClO could burst VLPs (Fig. 6B). It appeared that acid–ethanol also destroyed VLPs, but the VLPs did not bust. In addition, 70% ethanol appeared to have no effect on the morphological change of VLPs (Fig. 6B). Taken together, these data suggest that acid–ethanol acts directly on the capsid proteins of HuNoV and shows a virucidal effect against HuNoVs.

### Virucidal activity of commercially available alcohol-based disinfectants against HuNoVs

**Figure 7:**
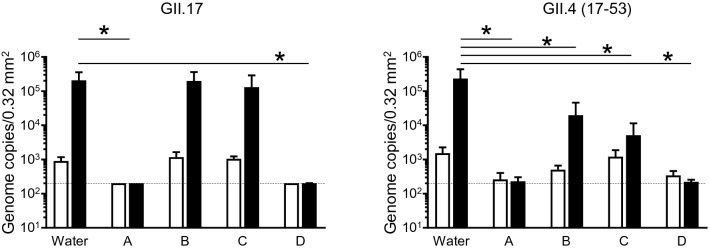
Inactivation of HuNoVs by commercially available acid–ethanol disinfectants in suspension. The solutions of the indicated HuNoV genotypes were suspended in ninefold volumes of four commercially available low-pH ethanol-based disinfectants (Products **A**–**D**) for 5 min. Inoculation and sampling were performed as in Fig. 1. Each value is representative of at least three independent experiments and is shown as the mean ± SD from 4–6 wells of supernatants for each culture group. *, *P* ≤ 0.05; **, *P* ≤ 0.01; ***, *P* ≤ 0.005; ****, *P* ≤ 0.001. Dashed lines represent the limit of detection.

We further assessed four commercially available alcohol-based disinfectants (containing 63% [v/v]–81% [v/v] ethanol) with a low pH (2.9–4.2) used for cookware or hand-sanitizers in Japan, all of which have been reported to have a virucidal effect on non-enveloped viruses. Among them, nine volumes of products A and D completely inactivated both GII.4 and GII.17 HuNoV genotypes after 5 min of incubation at room temperature (Fig. 7). Interestingly, under the same conditions, products B and C failed to inactivate these HuNoVs, even GII.4, which is inactivated by 70% ethanol alone (Fig. 1A).

Finally, we performed experiments to determine whether 100-fold dilution of all the disinfectants analyzed in this study would be sufficient for the attenuation of their cytotoxicity for IECs used for virus propagation (i.e., cytotoxicity control) and would also be able to neutralize their virucidal activity toward GII.17 HuNoV (i.e., neutralization control). As shown in Supplementary Fig. 1, 100-fold dilution of all disinfectants resulted in the appropriate neutralization of both cytotoxicity (Supplementary Fig. 1A,B) and virucidal activities (Supplementary Fig. 1C,D).

Taken together, the findings presented in this study suggested that the virucidal effect of each disinfectant against HuNoVs should be validated using an in vitro propagation system.

## Discussion

In 2016, Estes and her colleagues first reported that several HuNoV genotypes could propagate in IECs prepared from human small intestines^3^. Thereafter, our group also reported that human iPSC-derived IECs could be used for the cultivation of HuNoVs^4^. In this study, we investigated whether alcohol-based disinfectants have actual virucidal effects on several genotypes of HuNoV using our in vitro HuNoV cultivation system by analyzing its propagative potency as an index.

Using human tissue-derived IECs, Vinjé and his colleagues first examined the efficacy of ethanol and isopropanol in the reduction of the virus infectivity of GII.4 HuNoV and reported that the replication levels of GII.4 HuNoV incubated with tenfold volumes of 70% ethanol for 5 min were significantly but not completely lower compared with non-treated viruses, whereas incubation with 70% isopropanol had no effects^11^. Importantly, titers of input genomic RNA from HuNoV treated with 70% ethanol were severely reduced compared with the level of replication^9,11^. However, our results in this study were partially inconsistent with their reports. Although neither threefold volumes of 70% ethanol nor isopropanol could reduce the replication levels of GII.3, GII.6, GII.17, and GI.7 HuNoVs, the replication of two batches of GII.4 was almost completely inhibited (Fig. 1). One possible explanation for this discrepancy is that we used more diluted fecal filtrates for the experiments to modulate the genome copies of HuNoV to be 2 × 10^6^/well. Therefore, it is possible that more organic substances, which can interfere with the virucidal ability of 70% alcohol, were contained in the virus solutions in the previous report^11^. In fact, 3–5% beef extract contamination prevented the virucidal effects of acid–ethanol on GII.4 HuNoV in our system (Fig. 5).

Acidification is one of the inactivation methods for foodborne viruses. For example, rotaviruses lose their infectivity when incubated with a pH 2.0 solution^14^. Although feline calicivirus is also inactivated by incubation with a pH 2.0 solution for 30 min at 37 °C^15,16^, murine norovirus is stable under the same condition^8,16^. HuNoV (GII.17 genotype) appears to be resistant to low pH conditions since it can be propagated in IECs after 5 min incubation with 2% citric acid (pH 1.9) (Fig. 2A), suggesting that the capsid protein of HuNoV is more similar to that of murine norovirus than feline calicivirus. Nevertheless, we revealed that the addition of 1% citric acid to 70% ethanol or isopropanol dramatically increased its virucidal effect against HuNoVs (Fig. 2). The virucidal effect of acid-alcohols on other non-enveloped viruses^13,17^, including murine norovirus and feline calicivirus^6^, has been reported. Although murine norovirus and feline calicivirus are resistant to low-pH and alcohol, respectively, both are sensitive to acid-alcohol. From this point of view, HuNoVs exhibit both characteristics since they were stable in low pH alone and alcohol alone conditions. Given that noroviruses are a group of non-enveloped and hydrophilic RNA viruses, the mechanism underlying the virucidal effect of alcohol on HuNoVs likely involves protein denaturation rather than de-lipidation. This may also explain why either 70% ethanol or isopropanol alone was capable of inhibiting the replication of GII.4 HuNoV. The capsid protein of GII.4 genotype may be more sensitive to the protein denaturing effects of alcohol compared with other genotypes of HuNoV.

The alkaline-alcohol but not alkaline solution with a pH of 11 also showed a virucidal effect on HuNoVs, although it was milder than acid-alcohol (Fig. 4). It was previously reported that feline calicivirus is inactivated by both a high pH and low-pH solution^15^. Given that murine norovirus is sensitive to 70% alcohol, whereas feline calicivirus is sensitive to the non-neutral pH range, it is possible that a change in ionic and/or hydrogen bonds between alcohol and water is needed for 70% alcohol to exert its virucidal effect on HuNoVs, except for the GII.4 genotype. Soil containing stool and vomit would also affect the hydrate condition of alcohol, leading to a decrease in its denaturation effects on capsid proteins. In fact, a 3% beef extract solution almost completely inhibited the virucidal activity of acid–ethanol (Fig. 5). These unexpected effects apply for inert ingredients such as moisturizers and swelling agents, present in commercially available hand-sanitizers. It might be explained by the fact that some commercially available acid–ethanol disinfectants did not show a virucidal effect on HuNoVs, including GII.4 (Fig. 7). Therefore, whether the final product of disinfectants, including acid-alcohol-based ones, has an actual virucidal effect on HuNoVs in soil load conditions should be considered.

Because the electrolyte solution (i.e., MgSO_4_) used in this study, has been known to precipitate proteins via the effects of ions on biological and chemical processes in solutions, the addition of such salts (ions) could remove proteins adhering to virus surfaces. The order of the above effects is known as the Hofmeister series^18^, and both Mg^2+^ and SO_4_^2-^ are one of the strongest cations and anions, respectively. In addition, citrate ions are higher than phosphate ions and SO_4_^2-^ in the Hofmeister series^19^. Given that HuNoVs are usually excreted in feces and vomit containing various amounts of proteins, acid-alcohol disinfectants containing citric acid would be preferable.

Unlike murine norovirus and feline calicivirus, the genomic RNA of HuNoVs, especially the GII.17 genotype, was stable when the virus solution was incubated with 70% ethanol or acid–ethanol (Fig. 6A). Electron microscopic analysis revealed that acid–ethanol and NaClO could change the morphology of GII.17 VLPs similar to “apoptosis” and “necrosis” observed in animal cells, respectively. These findings also suggest that acid–ethanol could denature the capsid protein of HuNoVs, leading to a loss of their ability to infect IECs, whereas it had no impact on genomic RNA.

The significant advantage of acid-alcohol disinfectants is their good biological safety profiles as they can be generated using alcohol and some food additives. Thus, mineral salts, including acid-alcohols, could be used as HuNoV disinfectants for tableware, cookware, hands, and even foods. However, since it is difficult to evaluate the cytopathic effects of HuNoVs on IECs in our system, it is also challenging to determine the Median Tissue Culture Infectious Dose (TCID_50_) for HuNoVs to assess their virucidal activity. Therefore, future studies are required to develop standard guidelines for the determination of the virucidal activity of HuNoVs.

## Materials and methods

### Preparation of disinfectants

UltraPure DNase/RNase-Free Distilled Water (Thermo Fisher Scientific), ethanol, isopropanol, citric acid monohydrate, magnesium sulfate heptahydrate, sodium hydrogen carbonate, sodium carbonate (all purchased from Nacalai Tesque, Japan), and concentrated lemon juice (Pokka Sapporo Food and Beverage Ltd., Japan) were used to prepare the indicated disinfectants. Commercially available disinfectants and bleach (6% sodium hypochlorite) were purchased from a local retail store. After preparation, solutions were sterilized with 0.22 μm filters.

### Cells

The differentiation of human iPSCs into IECs and their culture were performed as described previously^4,20^. Each conditioned medium (CM) was prepared as described previously^20^. For HuNoV inoculation, dissociated IECs were seeded on 2.5% Matrigel-coated 96-well plates at 2 × 10^4^ cells/well in 100 μL of organoid culture medium (Advanced Dulbecco’s modified Eagle medium/F12 [Thermo Fisher Scientific] supplemented with 10 mM HEPES [pH 7.3; Thermo Fisher Scientific]; 2 mM Glutamax [Thermo Fisher Scientific]; 100 units/mL penicillin plus 100 μg/mL streptomycin; 25% mouse Wnt3a, human R-spondin1, and human Noggin [WRN] CM; 1 × B-27 [Thermo Fisher Scientific]; 50 ng/mL mouse epidermal growth factor [EGF, Peprotech]; 50 ng/mL human hepatocyte growth factor [R&D Systems]; 10 μM SB202190 [Sigma-Aldrich]; and 500 nM A83-01 [Tocris] plus 10 μM Y-27632 [Wako]). After 2 days of culture in a 5% CO_2_ incubator at 37 °C, the medium was changed to differentiation medium (Advanced Dulbecco’s modified Eagle medium/F12 supplemented with 10-mM HEPES [pH 7.3]; 2 mM Glutamax; 100 units/mL penicillin plus 100-μg/mL streptomycin; 1 × B-27; 12.5% human R-spondin1 and human Noggin [RN] CM; 50 ng/mL mouse EGF; and 500 nM A83-01). After another 2 days, the medium was changed to differentiation medium with 0.03% porcine bile (Sigma-Aldrich). The cells were incubated for another 2 days and then used for subsequent experiments.

### HuNoV preparation and infection

All virus samples used in this study were chosen from the HuNoV-positive stool specimens collected from Osaka prefecture, Japan during the 2016–2018 endemic season. Preparation and infection of HuNoV were done as described previously^4,21^. Briefly, HuNoV-positive stools were suspended in phosphate-buffered saline (PBS) at 10% (w/v) by vigorous vortexing. The suspensions were centrifuged at 12,000*g* for 30 min, and the supernatants were serially filtered with 0.45 μm and 0.22 μm filters. The filtered samples were aliquoted and stored at − 80 °C as an undiluted virus solution (see Table 1 for strain details). Just before use, each virus solution was diluted to 8 × 10^5^ (GII.6), 7.2 × 10^6^ (GI.7), or 8 × 10^6^ (other genotypes) genome equivalents/µL for a 1:3 suspension or 2 × 10^7^ genome equivalents/µL for a 1:9 suspension with PBS. In the case of soil load experiments, a solution of 12% (w/v) beef extract (Nacalai Tesque) was used for virus dilutions, which contained a final 3% or 5% beef extract. The virucidal suspension assays were performed using the similar method as that of the ASTM E1052-11. Four microliter of diluted virus solution were suspended in 12 µL (for 1:3) or 36 µL (for 1:9) of each disinfectant solution for the indicated times at room temperature. Then, each suspension was subjected to a 100-fold dilution with base medium (Advanced Dulbecco’s modified Eagle medium/F12 supplemented with 10 mM HEPES [pH 7.3], 2 mM Glutamax, and 100 units/mL penicillin plus 100 μg/mL streptomycin). The prepared IECs (3–6 wells per sample) were inoculated with 100 μL (2 × 10^6^ genome equivalents) of pre-treated virus solutions and then incubated for 3 h in a 5% CO_2_ incubator at 37 °C. The inoculum was then removed, and the cells were washed twice with 150 μL base medium. One hundred microliters of differentiation medium with 0.03% bile were added to the cells, which were then pipetted lightly twice and collected. This step was performed again, and the samples were collected as 3 h post-infection (hpi) reference samples (total 200 μL). Another 100 μL of differentiation medium with 0.03% bile were added into each well, and the mixtures were then cultured for 72 h in a 5% CO_2_ incubator at 37 °C. The supernatants were collected with one wash in the same way as 3 hpi reference samples (total 200 μL).

**Table 1:** List of HuNoVs used in this study.

Strain designation	Genotype variant	Titer (genome eq./μL)	Inoculation (genome eq./well)
GI.7	GI/Hu/JP/2018/GI.7[P7]/OsakaFB1836	7.2 × 10^6^	1.8 × 10^6^
GII.3	GII/Hu/JP/2016/GII.3[P12]/OsakaFB1650	4.3 × 10^7^	2.0 × 10^6^
GII.4 17–53	GII/Hu/JP/2017/GII.4 Sydney[P31]/OsakaFB1753	2.2 × 10^7^	2.0 × 10^6^
GII.4 17–231	GII/Hu/JP/2017/GII.4 Sydney[P31]/OsakaFB17231	8.3 × 10^7^	2.0 × 10^6^
GII.6	GII/Hu/JP/2018/GII.6 [P7]/OsakaFB1878	9.3 × 10^5^	2.0 × 10^5^
GII.17	GII/Hu/JP/2016/GII.17 [P17]/OsakaFB16421	5.8 × 10^7^	2.0 × 10^6^

To assess “virus control” and “neutralization control,” we used distilled water and 100-fold dilutions of each disinfectant, respectively, to substitute for the full-strength disinfectant solution. For the “cytotoxicity control,” cells were pretreated with 100-fold dilutions of each disinfectant and incubated for 3 h at 5% CO_2_ and 37 °C. After washing the cells twice with the base medium, the pretreated cells were inoculated with 100 μL (2 × 10^6^ genome equivalents) of GII.17 HuNoV followed by incubation for 3 h. Subsequent washing, culture, and sampling steps were performed as described above.

### Quantification of virus genome equivalents

A PureLink Viral RNA/DNA Mini Kit (Invitrogen) was used to prepare RNA from diluted virus solutions and samples collected at 3 and 72 hpi. RT-qPCR was performed using a qPCR Norovirus (GI/GII) Typing Kit (TaKaRa) and LightCycler 480 System (Roche) following the manufacturers’ protocols.

### Statistical analysis

Results were compared using either unpaired two-tailed Student’s *t*-test or one-way ANOVA followed by Dunnett’s multiple comparisons test. Statistical significance was established at *P* < 0.05. All statistical analyses were conducted using GraphPad Prism 7.

### Ethics statement

Under the informed consent with providing a means to opt out, all stool samples were collected and provided by the Osaka Institute of Public Health. The study was performed in accordance with the relevant guidelines and was approved by the Human Ethical Committee of Research Institute for Microbial Diseases, Osaka University (approval #28-3) and the Osaka Institute of Public Health (approval #1602-04-2).

## Supplementary information

Supplementary Information
