# Supplementary material for: Alcohol abrogates human norovirus infectivity in a pH-dependent manner
Source: Sci Rep. 2020 Sep 28;10:15878. doi: 10.1038/s41598-020-72609-z (PMC7522253; doi:10.1038/s41598-020-72609-z)
Supplement: Supplementary file 1 — Supplementary Information [file 41598_2020_72609_MOESM1_ESM.pdf]

Alcohol abrogates human norovirus infectivity in a pH-dependent manner

Shintaro Sato[1,2,3#], Naomi Matsumoto[1,2], Kota Hisaie[1], and Satoshi Uematsu[3]

[1] Mucosal Vaccine Project, BIKEN Innovative Vaccine Research Alliance Laboratories, Research Institute for Microbial Diseases, Osaka University, Osaka 565-0871, Japan.

[2] Department of Virology, Research Center for Infectious Disease Control, Research Institute for Microbial Diseases, Osaka University, Osaka 565-0871, Japan.

[3] Department of Immunology and Genomics, Osaka City University Graduate School of Medicine, Osaka 545-8585, Japan.

[#]Corresponding authors:

Shintaro Sato

Telephone No.: +81-6-6877-4781

Fax No.: +81-6-6877-4786

E-mail address: shinta@biken.osaka-u.ac.jp

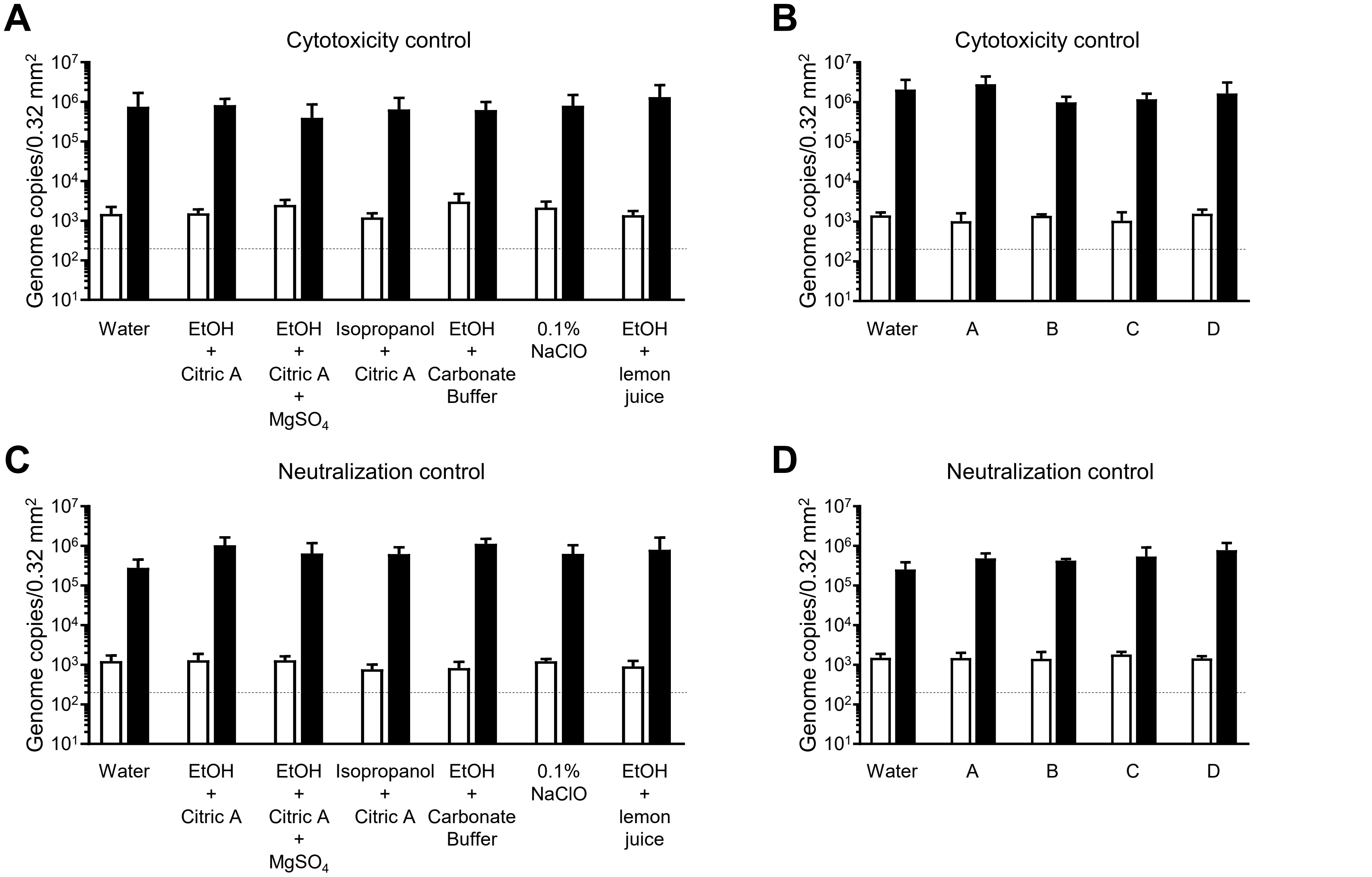

Supplementary Figure 1: Cytotoxicity and neutralization control of disinfectants using in this study

(*A* and *B*) Cytotoxicity check of indicated disinfectants. Mono-layered human iPSC-derived IECs were pretreated with 100-fold diluted disinfectants for 3 h. After the washing out, the cells were inoculated with 2×10^6 genome equivalents of GII.17 HuNoV for 3 h. After the washing out and taking 3 hpi samples (open bars), cells were further cultured for 72 h. The 72 hpi samples (closed bars) were corrected, and then genome equivalents were quantified with RT-qPCR. (*C* and *D*) Neutralization control experiments for indicated disinfectants. Two millions genotype equivalents of GII.17 HuNoV (one part) were incubated with neutralization control solution (nine parts), which contained 100-fold diluted each disinfectant, for 5 min at room temperature. Inoculation and sampling were performed as described in the Materials and Methods. Each value is representative of two independent experiments and is shown as the mean ± SD from 4 wells of supernatants for each culture group.
